# Trends and outcome of statin therapy in dialysis patients with atherosclerotic cardiovascular diseases: A population-based cohort study

**DOI:** 10.1371/journal.pone.0286670

**Published:** 2023-06-02

**Authors:** Myunhee Lee, Yu Ah Hong, Jun-Pyo Myong, Kyusup Lee, Mahn-Won Park, Dae-Won Kim

**Affiliations:** 1 Division of Cardiology, Department of Internal Medicine, Daejeon St. Mary’s Hospital, The Catholic University of Korea, Seoul, Korea; 2 Catholic Research Institute for Intractable Cardiovascular Disease CRID, College of Medicine, The Catholic University of Korea, Seoul, Korea; 3 Division of Nephrology, Department of Internal Medicine, Daejeon St. Mary’s Hospital, The Catholic University of Korea, Seoul, Korea; 4 Department of Occupational and Environmental Medicine, Seoul St. Mary’s Hospital, The Catholic University of Korea, Seoul, Korea; Dartmouth Health, UNITED STATES

## Abstract

**Background:**

Although statins are an effective strategy for the secondary prevention of atherosclerotic cardiovascular disease (ASCVD) in the general population, the benefits for dialysis patients are controversial. We sought to assess trends of statin use and evaluate outcomes of statin therapy in dialysis patients with different types of ASCVD.

**Methods:**

This nationwide retrospective population-based cohort study using data from the Korean National Health Insurance Service included adult patients (aged ≥ 18 years) undergoing chronic dialysis who had an initial ASCVD event in the time period of 2013 to 2018. Annual trends of statin use according to age, sex, and ASCVD types were analyzed. The association between 1-year mortality and statin use was examined using multivariable Cox proportional hazards regression analyses.

**Results:**

Among 17,242 subjects, 9,611(55.7%) patients were statin users. The overall prevalence of statin use increased from 52.9% in 2013 to 57.7% in 2018; the majority (77%) of dialysis patients were prescribed moderate-intensity statins. The proportions of low- or moderate-intensity statin use were similar, but high-intensity statin use increased from 5.7% in 2013 to 10.5% in 2018. The use of the statin/ezetimibe combination has gradually increased since 2016. Statin use was independently associated with the reduced 1-year all-cause mortality after adjusting for confounding factors (hazard ratio [HR] 0.89, 95% confidence interval [CI] 0.80–0.96, *P* = 0.004).

**Conclusion:**

The prevalence of statin prescriptions in dialysis patients after ASCVD event increased from 2013 to 2018. Most patients received moderate-intensity statin. However, high-intensity statin and statin/ezetimibe combination therapy has remarkably increased. Statin use was associated with decreased 1-year all-cause mortality in dialysis patients with ASCVD.

## Introduction

Cardiovascular (CV) disease is a major health care concern for patients with end-stage renal disease (ESRD) due to higher rates of morbidity and mortality compared to the general population with normal renal function [1]. According to the 2019 Korean Renal Data System annual report, CV mortality was the leading cause of death in ESRD over 20 years, accounting for 45.0–54.0% of deaths in Korea [2]. Chronic kidney disease (CKD) is considered to be an independent risk factor for the development of atherosclerotic cardiovascular disease (ASCVD) [3]. Moreover, statins are an effective strategy for the secondary prevention of ASCVD in the general population at a high risk of CV disease and in patients with preexisting ASCVD [4]. Several previous trials and meta-analyses have shown similar results in patients with nondialysis-dependent CKD [5, 6]. However, the beneficial effects of statins cannot be generalized to ESRD patients because previous landmark randomized controlled trials (RCTs) involving patients on dialysis failed to improve ASCVD outcomes [7–9].

Based on this evidence, The Kidney Disease: Improving Global Outcomes (KDIGO) 2013 guideline suggests neither initiating nor discontinuing statins in patients at or after dialysis initiation for the primary prevention of CV disease, and this guideline did not offer specific recommendations on secondary prevention of CV events in dialysis patients [10]. Consistent with the KDIGO guideline, the 2018 American College of Cardiology/American Heart Association/Multisociety (ACC/AHA/MS) and the 2019 European Society of Cardiology (ESC) and European Atherosclerosis Society (EAS) guidelines do not recommend the initiation of statins for adults requiring dialysis who are free of ASCVD, based on a lack of CV benefit; however, these guidelines do not provide a clear answer for the statin therapy in chronic dialysis patients who experience new ASCVD events [11, 12]. The current guidelines highlight the uncertainty about the benefits of statins in patients on dialysis because the data is inconsistent for the efficacy of statin therapy, and limited data for trends of statin use and outcomes are available in patients on dialysis with ASCVD.

Therefore, the objective of this study was to (1) investigate the annual trends of statin prescribing patterns in patients on chronic dialysis with new ASCVD events and (2) evaluate whether statin treatment is associated with improved 1-year mortality in these populations.

## Methods

### Data source and study participants

This nationwide retrospective population-based cohort study was performed by using data from the Korea NHIS (National Health Insurance Service) database, which is an obligatory single-payer health insurance system that offers medical care coverage for up to 98% of the Korean population [13]. The NHIS database includes demographic data on the beneficiaries, procedures, prescription records of all of the medical services, diagnostic codes defined by the International Classification of Diseases 10th Revision (ICD-10), and complete mortality data.

**Fig 1:**
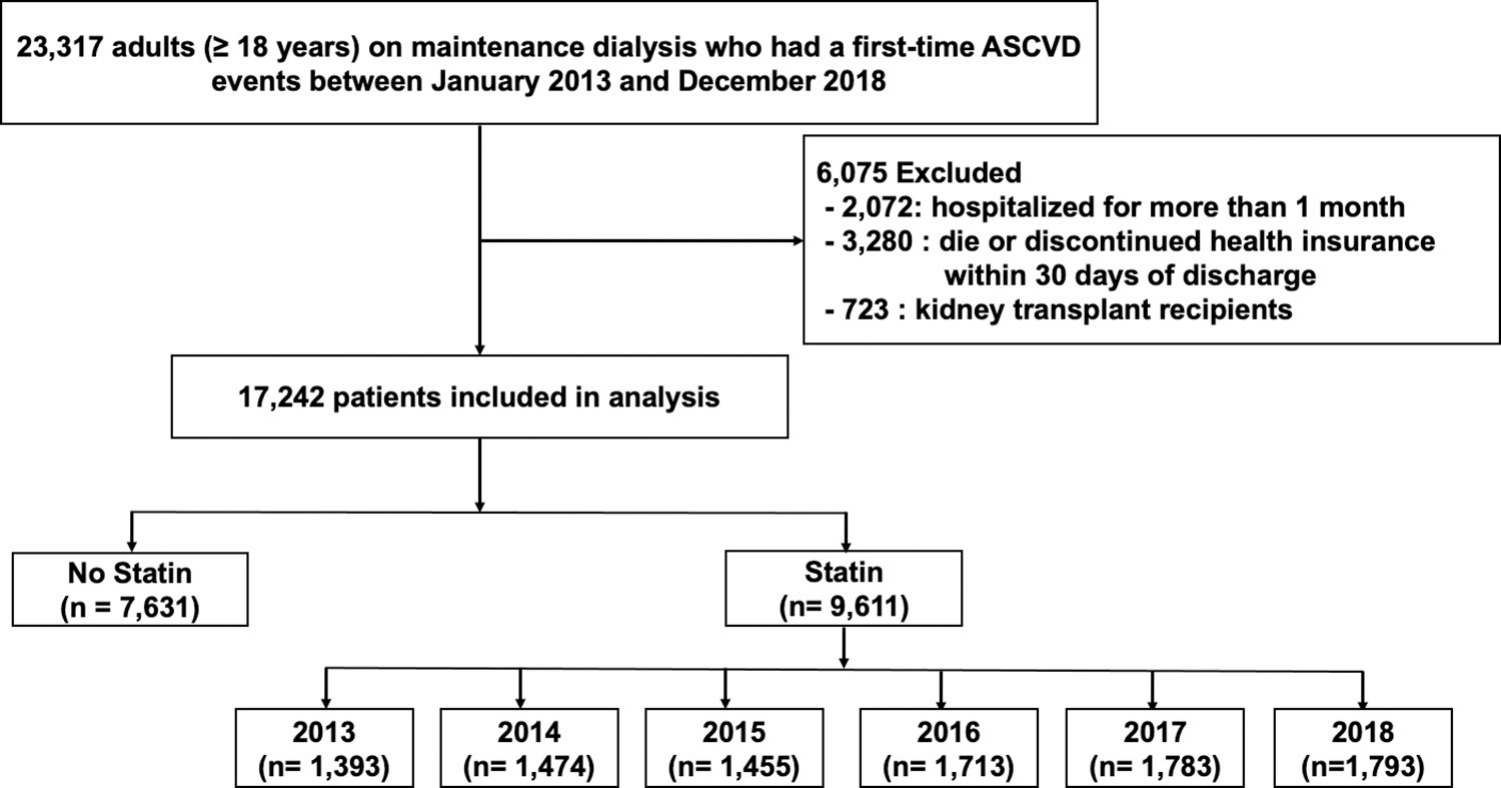
Flow chart of the study design. ASCVD, atherosclerotic cardiovascular disease.

We initially recruited ESRD patients aged ≥ 18 years who had their first ASCVD event between January 2013 and December 2018 and who were receiving maintenance renal replacement therapy (hemodialysis or peritoneal dialysis) before the ASCVD event. To assess the trends of statin use, adherence, and outcomes among patients on dialysis after experiencing a new ASCVD event, we excluded patients with preexisting ASCVD. This exclusion was based on the absence of any inpatient, outpatient, or emergency room ASCVD claims during the six months leading up to the date when they entered the cohort. The index date was defined as the date when the patient experienced a first-time ASCVD event. If a patient had an admission, the index date was defined as the discharge date. Patients were excluded if they died or discontinued health insurance within 30 days of discharge, had a record of hospital stays longer than one month at the index hospitalization, or received kidney transplantation before the index date. A total of 17,242 adult ESRD patients who had their first ASCVD event were finally included in the analysis. The inclusion and exclusion criteria of the study population are summarized in **Fig 1**. Patient baseline characteristics were reported on the index date. The approval by the Institutional Review Board (IRB) of each participating institution (IRB of Daejeon St. Mary’s Hospital, IRB of Seoul St. Mary’s Hospital) and the requirement for informed consent were waived because the data analyses were retrospectively performed using anonymized data derived from the Korea NHIS database (NHIS-2021-1-523).

### Definitions

Patients with ESRD undergoing maintenance renal replacement therapy were defined as those who met the following criteria: (1) ICD-10 codes related to CKD/ESRD (N18-19) and (2) procedure codes related to hemodialysis or peritoneal dialysis for at least three months. ASCVD events were categorized into one of the following groups: coronary heart disease (CHD) (myocardial infarction [MI], angina, and coronary revascularization), cerebrovascular accident (CVA) (ischemic stroke and transient ischemic attack [TIA]), or peripheral artery disease (PAD) [4]. The diagnosis of ASCVD was made if the ICD-10 code for each ASCVD component was recorded with a history of admission/emergency department visits at least once or an outpatient clinic visit more than twice. Patients with angina, ischemic stroke, TIA, or PAD were required to have both ICD-10 codes for each diagnosis and additional claim codes, such as imaging studies, endovascular procedures, or surgery, solely to include patients who had clinical evidence of ASCVD. We excluded patients with ischemic stroke or TIA if they had a diagnosis of atrial fibrillation to exclude the cardioembolic origin of stroke. Coronary revascularization was identified by using related procedure codes. Detailed information and definitions used in this study are provided in **S1 Table**.

### Outcome measures

Patients were followed until December 2019 or until the date of an exemption from health insurance services or death (whichever event happened first). The primary outcomes were all-cause mortality and cardiac mortality during the 12 months after the index date. The information regarding all-cause and cardiac mortality of the study participants was extracted from the death certificate database provided by Statistics Korea. Cardiac mortality was defined as the specific cause of death according to the ICD-10 codes: I00-I99. This data was matched with the medical claim data through Health Insurance and Review Assessment. Statin use was defined as at least one prescription recorded in claim data within 30 days of the index date. The Charlson Comorbidity Index (CCI) was calculated to assess patients’ underlying medical conditions [14]. Adherence to statin therapy was assessed via the proportion of days covered (PDC) at the end of 1 year. The PDC was calculated using the following equation: number of days covered by any statin prescription during one year after index date/365. Statin intensities were categorized into high-intensity, moderate-intensity, and low-intensity according to the 2013 ACC/AHA guidelines [4].

### Statistical analysis

Continuous variables are expressed as mean ± SD or median (interquartile range), while categorical variables are presented as absolute values and percentages. Differences between groups of categorical variables were analyzed via the chi-square or Fisher exact test. Differences between groups or continuous variables were analyzed using a two-tailed Student’s t-test or the Mann-Whitney U test. Jonckheere-Terpstra test for continuous dependent variables and Cochran Armitage test for dichotomous dependent variables were used for trend analysis. The trends were assessed in the overall population and each subgroup based on a patient’s initial ASCVD event. Subgroup analyses were performed according to age group (ages < 65 years, 65–74 years, and ≥ 75 years) and sex. A multivariable Cox proportional hazards regression model was used to assess the association between mortality and statin use, adjusted for age, sex, index year, and comorbidities. A two-sided P < 0.05 was considered to be statistically significant. Statistical analyses were performed using SAS software V.9.4 (SAS Institute Inc., Cary, NC, USA).

## Results

### Baseline characteristics of the study population

**Table 1** shows the baseline characteristics of the study population according to enrollment year. The mean age of the total population was 63.0 ± 11.3 years, and 10,650 patients (61.8%) were male. Overall, 9,611 patients (55.7%) were prescribed statin therapy after a first-time ASCVD event. The mean age of the patients on dialysis with a first-time ASCVD event decreased from 64.2 ± 10.7 years in 2013 to 62.1 ± 11.7 years in 2018 (*P* for trend < 0.001). Moreover, diabetes with chronic complications, chronic pulmonary disease, and cancer gradually increased in patients on dialysis with ASCVD. During the study period, the number of patients with PAD was significantly increased, whereas the number of patients with CHD or CVA was significantly decreased (all *P* < 0.001).

**Table 1:** Baseline characteristics of total study participants from 2013 to 2018.

	2013 (n = 2633)	2014 (n = 2636)	2015 (n = 2594)	2016 (n = 3078)	2017 (n = 3194)	2018 (n = 3107)	*P* for trend
Age (year)	64.2 ± 10.7	63.9 ± 10.8	63.3 ± 11.1	62.3 ± 11.7	62.7 ± 11.5	62.1 ± 11.7	<0.001
Sex (male, %)	1624 (61.7)	1630 (61.8)	1574 (60.7)	1911 (62.1)	1991 (62.3)	1920 (61.8)	0.865
**Comorbidities (n, %)**							
Diabetes	1912 (72.6)	1865 (70.6)	1768 (68.2)	2116 (68.8)	2163 (67.7)	2076 (66.8)	<0.001
Diabetes with chronic complications	1746 (66.3)	1783 (67.6)	1764 (68.0)	2103 (68.3)	2115 (66.2)	2050 (66.0)	0.221
Hyperlipidemia	1916 (72.8)	1979 (75.1)	2002 (77.2)	2368 (76.9)	2486 (77.8)	2494 (80.3)	< .0001
Hypertension	2498 (94.9)	2457 (93.2)	2426 (93.5)	2885 (93.7)	2973 (93.1)	2902 (93.4)	0.089
Congestive heart failure	1206 (45.8)	1206 (45.8)	1167 (45.0)	1467 (47.7)	1463 (45.8)	1439 (46.3)	0.454
Atrial fibrillation	236 (9.0)	232 (8.8)	260 (10.0)	288 (9.4)	324 (10.1)	315 (10.1)	0.302
Chronic pulmonary disease	953 (36.2)	990 (37.6)	978 (37.7)	1176 (38.2)	1267 (39.7)	1250 (40.2)	0.020
Moderate to severe liver disease	30 (1.1)	21 (0.8)	19 (0.7)	15 (0.5)	21 (0.7)	21 (0.7)	0.108
Cancer	238 (9.0)	242 (9.2)	260 (10.0)	336 (10.9)	396 (12.4)	381 (12.3)	<0.001
**ASCVD types (n, %)**							
CHD	1780 (67.6)	1811 (68.7)	1730 (66.7)	1969 (64.0)	1909 (59.8)	1821 (58.6)	<0.001
CVA	507 (19.3)	441 (16.7)	445 (17.1)	497 (16.1)	537 (16.8)	440 (14.2)	<0.001
PAD	346 (13.1)	384 (14.6)	419 (16.2)	612 (19.9)	748 (23.4)	846 (27.2)	<0.001

Data are mean ± SD or number (%).

ASCVD, atherosclerotic cardiovascular disease; CHD, coronary heart disease; CVA, cerebral vascular accident; and PAD, peripheral artery disease.

**S2 Table** shows the baseline characteristics according to ASCVD subtypes. Among 17,242 patients, 11,020 (63.9%) patients were first diagnosed with CHD, followed by PAD (3,355, 19.5%) and CVA (2,867, 16.6%). Patients with CVA were older than those with CHD or PAD (*P* < 0.001). Furthermore, a higher proportion of males were diagnosed with CHD than with CVA or PAD. Patients with CHD were more likely to have multiple comorbidities, such as hypertension, congestive heart failure, or chronic pulmonary disease, than patients with CVA or PAD, as demonstrated by a higher mean CCI. Additionally, adherence to statin therapy gradually increased from 2013 to 2018, especially adherence to statin therapy in patients with CHD, which significantly increased from 76.9% in 2013 to 86.0% in 2018 (*P* = 0.004, **S3 Table**).

### Annual trends of statin prescription in dialysis patients with ASCVD

**Fig 2:**
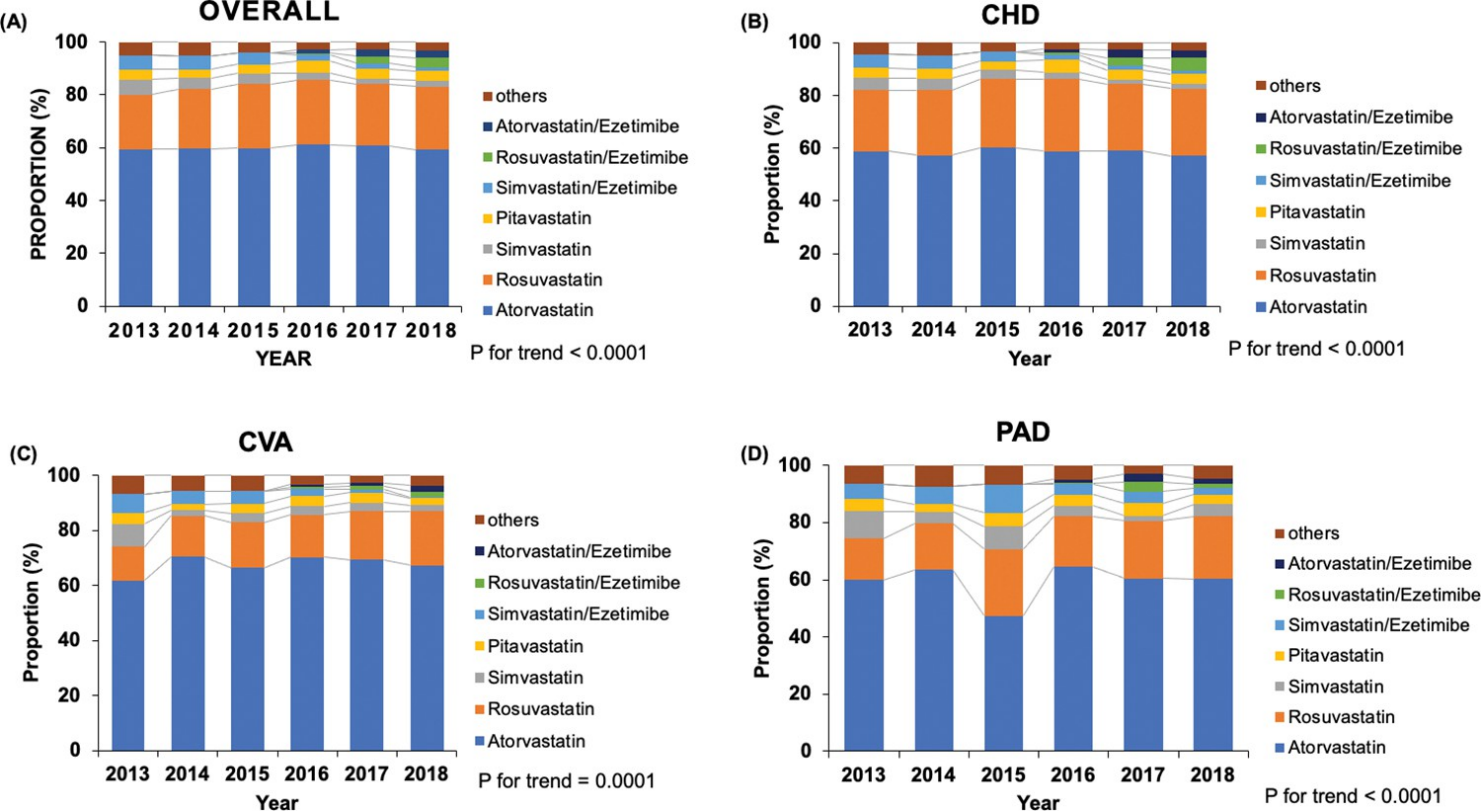
Trends of statin use stratified by brand-name statins and the ASCVD subtypes in patients on dialysis with ASCVD. (a) Overall, (b) CHD, (c) CVA, (d) PAD. ASCVD, atherosclerotic cardiovascular disease; CHD, coronary heart disease; CVA, cerebrovascular accident; PAD, peripheral artery disease.

**Table 2** shows the annual trends of statin prescription as stratified by ASCVD subtypes and statin intensity. The overall statin prescription rate significantly increased from 52.9% in 2013 to 57.7% in 2018 (*P* < 0.001). In patients with CHD or CVA, the proportion of statin prescriptions steadily increased from 2013 to 2018 (*P* < 0.001 and *P* = 0.012, respectively), whereas that in patients with PAD did not (*P* = 0.184). Approximately 80% of all statin users received moderate-intensity statins. In the overall population, low- and moderate-intensity statin prescription rates remained steady throughout the year, whereas high-intensity statin prescription rates increased from 5.7% in 2013 to 10.5% in 2018. A similar trend of statin prescription was observed in patients with CHD or CVA, but this trend was not observed in patients with PAD. Atorvastatin was the most widely prescribed statin during study period, followed by rosuvastatin. Moreover, the use of the statin/ezetimibe combination has substantially increased since 2016 (**Fig 2**).

**Table 2:** Trends of statin use stratified by ASCVD subtypes and statin intensity in dialysis patients from 2013 to 2018.

	2013 (n = 2633)	2014 (n = 2636)	2015 (n = 2594)	2016 (n = 3078)	2017 (n = 3194)	2018 (n = 3107)	*P* for trend
**Overall (n = 17458)**							
No statin use (n, %)	1240 (47.1)	1162 (44.1)	1139 (43.9)	1365 (44.4)	1411 (44.2)	1314 (42.3)	< .001
Statin use (n, %)	1393 (52.9)	1474 (55.9)	1455 (56.1)	1713 (55.6)	1783 (55.8)	1793 (57.7)	< .001
Low intensity	29 (1.1)	38 (1.4)	26 (1.0)	22 (0.7)	23 (0.7)	31 (1.0)	
Moderate intensity	1135 (43.1)	1146 (43.5)	1120 (43.2)	1340 (43.5)	1338 (41.9)	1297 (41.7)	
High intensity	150 (5.7)	212 (8.0)	244 (9.4)	279 (9.1)	288 (9.0)	325 (10.5)	
Statin + Ezetimibe	79 (3.0)	78 (3.0)	65 (2.5)	72 (2.3)	134 (4.2)	140 (4.5)	
**CHD (n = 11020)**							
No statin use (n, %)	744 (41.8)	674 (37.2)	636 (36.8)	736 (37.4)	670 (35.1)	618 (33.9)	< .001
Statin use (n, %)	1036 (58.2)	1137 (62.8)	1094 (63.2)	1233 (62.6)	1239 (64.9)	1203 (66.1)	< .001
Low intensity	23 (1.3)	25 (1.4)	15 (0.9)	15 (0.8)	15 (0.8)	20 (1.1)	
Moderate intensity	841 (47.2)	885 (48.9)	843 (48.7)	945 (48.0)	901 (47.2)	815 (44.8)	
High intensity	116 (6.5)	167 (9.2)	197 (11.4)	224 (11.4)	228 (11.9)	259 (14.2)	
Statin + Ezetimibe	56 (3.2)	60 (3.3)	39 (2.3)	49 (2.5)	95 (5.0)	109 (6.0)	
**CVA (n = 3083)**							
No statin use (n, %)	287 (56.6)	252 (57.1)	234 (52.6)	259 (52.1)	286 (53.3)	202 (45.9)	0.022
Statin use (n, %)	220 (43.4)	189 (42.9)	211 (47.4)	238 (47.9)	251 (46.7)	238 (54.1)	0.012
Low intensity	5 (1.0)	8 (1.8)	6 (1.4)	1 (0.2)	5 (0.9)	6 (1.4)	
Moderate intensity	178 (35.1)	137 (31.2)	162 (36.4)	194 (39.0)	193 (35.9)	175 (39.8)	
High intensity	22 (4.3)	35 (7.9)	33 (7.4)	33 (6.6)	44 (8.2)	46 (10.5)	
Statin + Ezetimibe	15 (3.0)	9 (2.0)	10 (2.3)	10 (2.0)	9 (1.7)	11 (2.5)	
**PAD (n = 3355)**							
No statin use (n, %)	209 (60.4)	236 (61.5)	269 (64.2)	370 (60.5)	455 (60.8)	494 (58.4)	0.178
Statin use (n, %)	137 (39.6)	148 (38.5)	150 (35.8)	242 (39.5)	293 (39.2)	352 (41.6)	0.184
Low intensity	1 (0.3)	5 (1.3)	5 (1.2)	6 (1.0)	3 (0.4)	5 (0.6)	
Moderate intensity	116 (33.5)	124 (32.3)	115 (27.5)	201 (32.8)	244 (32.6)	307 (36.3)	
High intensity	12 (3.5)	10 (2.6)	14 (3.3)	22 (3.6)	16 (2.1)	20 (2.4)	
Statin + Ezetimibe	8 (2.3)	9 (2.3)	16 (3.8)	13 (2.1)	30 (4.0)	20 (2.4)	

Data are number (%).

ASCVD, atherosclerotic cardiovascular disease; CHD, coronary heart disease; CVA, cerebral vascular accident; and PAD, peripheral artery disease

**Fig 3:**
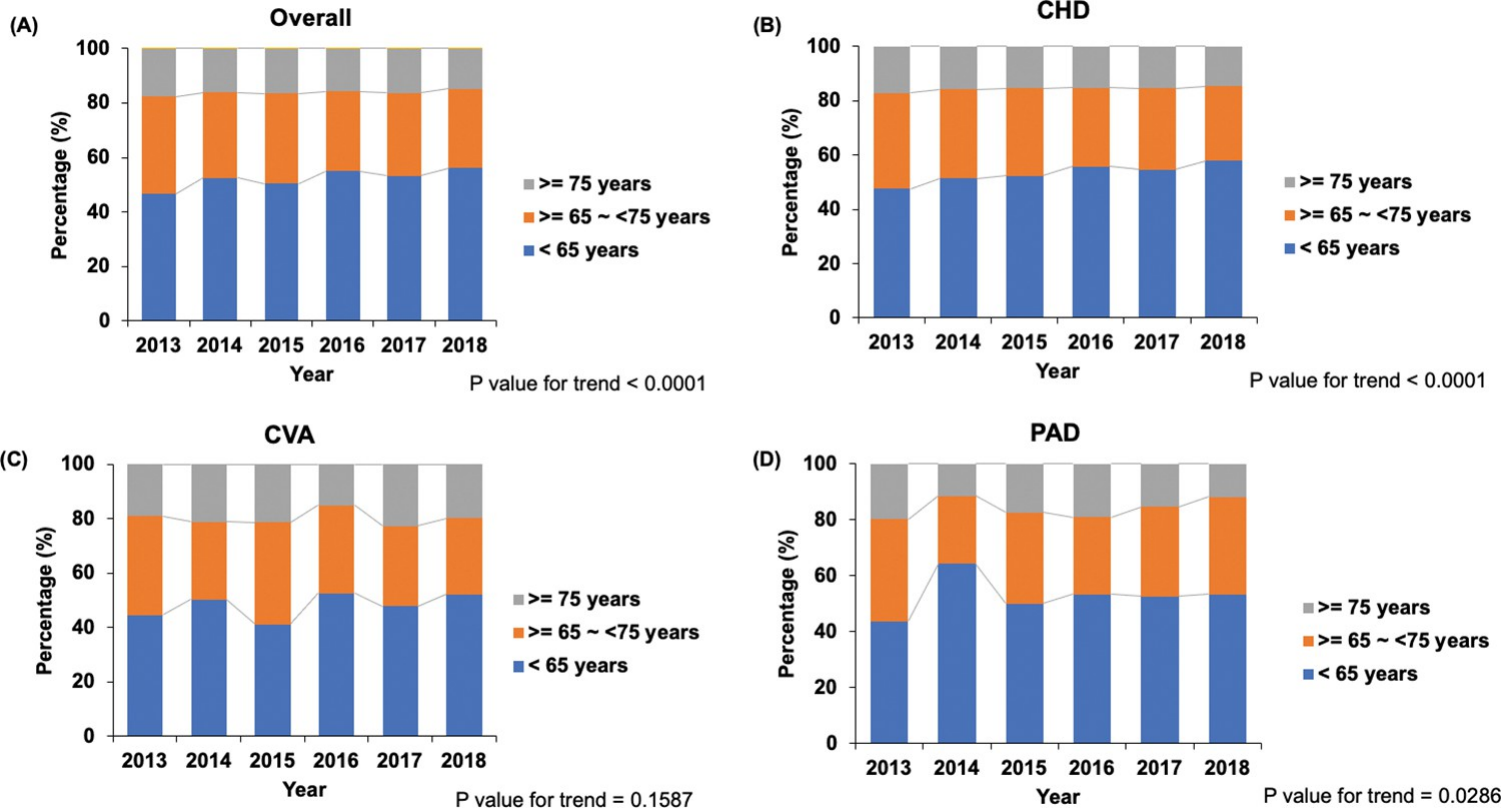
Trends of statin use stratified by age and the ASCVD subtypes in patients on dialysis with ASCVD. (a) Overall, (b) CHD, (c) CVA, (d) PAD. ASCVD, atherosclerotic cardiovascular disease; CHD, coronary heart disease; CVA, cerebrovascular accident; PAD, peripheral artery disease.

**Fig 4:**
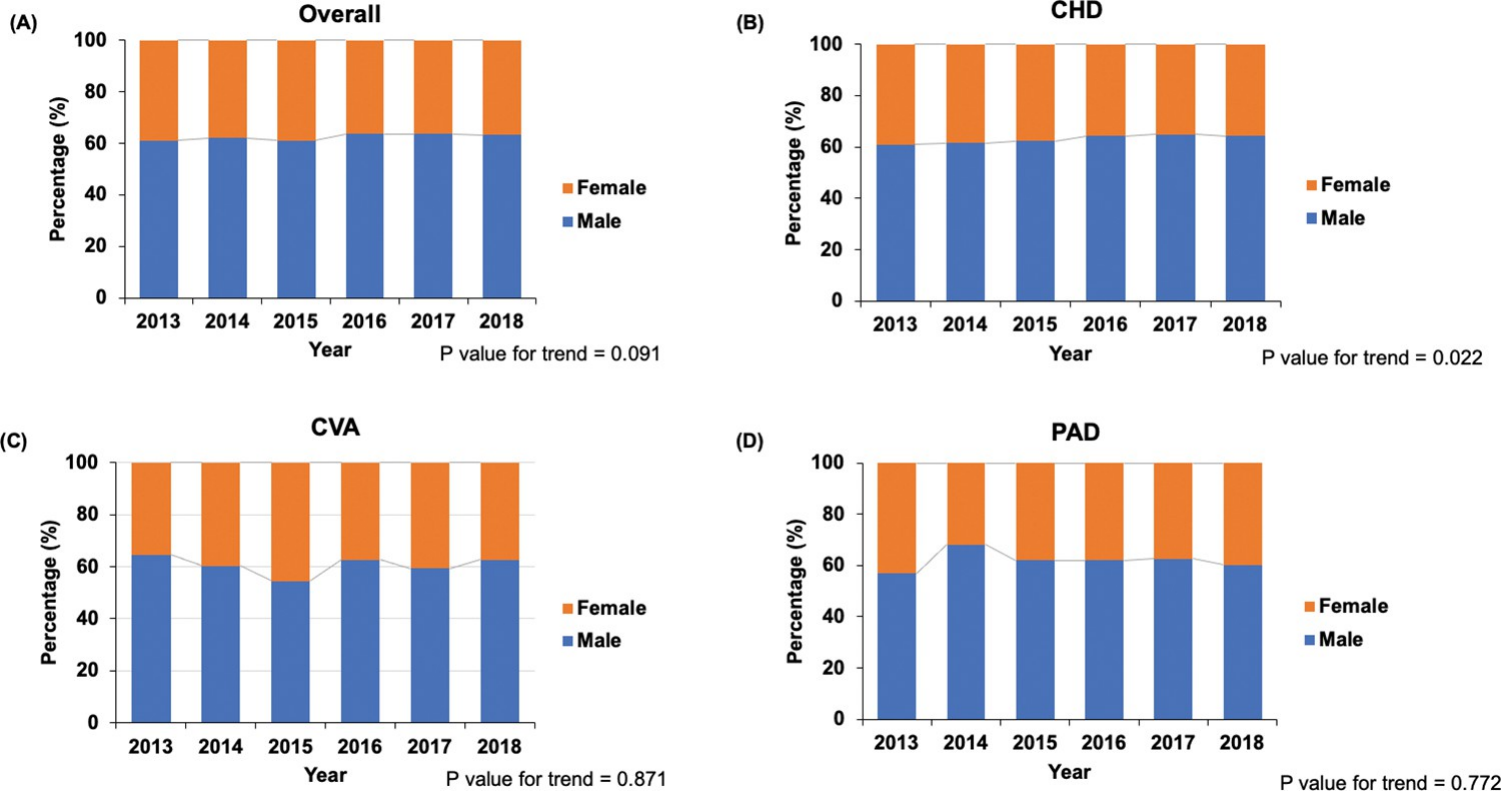
Trends of statin use stratified by sex and the ASCVD subtypes in patients on dialysis with ASCVD. (a) Overall, (b) CHD, (c) CVA, (d) PAD. ASCVD, atherosclerotic cardiovascular disease; CHD, coronary heart disease; CVA, cerebrovascular accident; PAD, peripheral artery disease.

The annual trends of the statin prescription rate stratified by age and sex are shown in **Figs 3** and **4**. When stratified by age group, dialysis patients aged < 65 years were more likely to receive statins than patients aged 65–74 years or ≥ 75 years, whereas patients aged ≥ 75 years were less likely to receive statins (*P* < 0.001). Among patients with CHD or PAD, the statin prescription rate gradually increased over the years in patients aged < 65 years (CHD: 47.5% in 2013, 57.9% in 2018; PAD: 43.8% in 2013, 53.4% in 2018), whereas it gradually decreased in patients aged ≥ 75 years (CHD: 17.1% in 2013, 14.6% in 2018; PAD: 19.7% in 2013, 11.9% in 2018) (**Fig 3**). Furthermore, women were less likely to receive statins than men, irrespective of ASCVD subtypes. In patients with CHD, stain use in men increased from 61% in 2013 to 64.2% in 2018, whereas statin use in women decreased from 39% in 2013 to 35.8% in 2018 (*P* = 0.022) (**Fig 4**).

### Associations of statin use with 1-year all-cause or cardiac mortality

During the 12 months of follow-up, statin use had a lower crude or age-and sex-adjusted all-cause mortality in the overall population (crude hazard ratio [HR]: 0.91, 95% confidence interval [CI]: 0.83–0.99, *P* = 0.034; age- and sex-adjusted HR: 0.90, 95% CI: 0.82–0.98, *P* = 0.020). In the multivariable Cox analysis adjusted for age, sex, CCI, and index year, statin use was independently associated with a reduced risk of all-cause mortality (HR: 0.89, 95% CI: 0.80–0.96, *P* = 0.004). When stratified by ASCVD subtypes, statin use was independently associated with a reduced risk of all-cause mortality in dialysis patients with CHD (HR: 0.88, 95% CI: 0.78–0.99, P = 0.037) and dialysis patients with CVA (HR: 0.74, 95% CI: 0.59–0.93, *P* = 0.009) in a fully adjusted model. However, no significant association was observed between statin use and all-cause mortality in dialysis patients with PAD (fully adjusted HR: 1.17, 95% CI: 0.97–1.41, *P* = 0.098) (**Table 3**). However, statin use was independently associated with an increased risk of cardiac mortality in the overall population (fully adjusted HR: 1.27, 95% CI: 1.01–1.59, *P* = 0.042). There were no significant associations between statin use and cardiac mortality in dialysis patients with CHD or CVA, whereas an increased risk of cardiac mortality for statin use was observed in dialysis patients with PAD (fully adjusted HR: 2.12, 95% CI: 1.17–3.86, *P* = 0.014) (**Table 3**).

**Table 3:** Association between statin use and all-cause or cardiac mortalities within 1 year in patients on dialysis with ASCVD.

	Unadjusted		Adjusted for age and sex		Fully Adjusted *	
	HR (95% CI)	*P* value	HR (95% CI)	*P* value	HR (95% CI)	*P* value
**All-cause mortality**						
No statin use	1 (reference)		1 (reference)		1 (reference)	
Statin use						
Overall	0.91 (0.83–0.99)	0.034	0.90 (0.82–0.98)	0.020	0.89 (0.80–0.96)	0.004
CHD	0.93 (0.83–1.05)	0.243	0.90 (0.80–1.01)	0.073	0.88 (0.78–0.99)	0.037
CVA	0.71 (0.57–0.89)	0.003	0.74 (0.59–0.92)	0.008	0.74 (0.59–0.93)	0.009
PAD	1.25 (1.04–1.50)	0.018	1.23 (1.03–1.48)	0.025	1.17 (0.97–1.41)	0.098
**Cardiac mortality**						
No statin use	1 (reference)		1 (reference)		1 (reference)	
Statin use						
Overall	1.26 (1.01–1.58)	0.041	1.25 (0.99–1.56)	0.055	1.27 (1.01–1.59)	0.042
CHD	1.10 (0.84–1.44)	0.480	1.05 (0.81–1.38)	0.707	1.05 (0.80–1.38)	0.709
CVA	0.97 (0.53–1.76)	0.912	0.99 (0.55–1.81)	0.982	1.04 (0.57–1.91)	0.897
PAD	2.04 (1.14–3.65)	0.017	2.01 (1.12–3.60)	0.019	2.12 (1.17–3.86)	0.014

* Adjusted for age, sex, comorbidity, CCI, index year.

ASCVD, atherosclerotic cardiovascular disease; CCI, charlson comorbidity index; CHD, coronary heart disease; CI, confidence interval; CVA, cerebral vascular accident; HR, hazard ratio; and PAD, peripheral artery disease.

## Discussion

To our knowledge, this is the first study to report annual trends in the prescription and outcomes of statin therapy for secondary prevention on dialysis with ASCVD in Korea. The overall prevalence of statin prescriptions gradually increased from 2013 to 2018, with most patients receiving moderate-intensity statins. The statin/ezetimibe combination therapy has remarkably increased since 2016. The proportions of low- or moderate-intensity statin use were similar, but high-intensity statin use approximately doubled over this time frame. The statin use was independently associated with a lower risk for 1-year all-cause mortality, especially in dialysis patients with CHD or CVA. However, statin use was associated with an increased risk for 1-year cardiac mortality, particularly in dialysis patients with PAD.

The trends of statin use in the general population showed that statin use for the secondary prevention of ASCVD had increased over time [15]. However, there has been little research on prescription trends or the impact of statins on secondary prevention in dialysis patients. In this study, we showed that statin use in dialysis patients with ASCVD events increased from 52.9% to 57.7% between 2013 and 2018. The use of statins in our study was 58.2% to 66.1% of dialysis patients with CHD, which was relatively lower than the average use (approximately 80%) of statins among the general population with CHD [15, 16]. We also found that older patients had a lower tendency to be prescribed statins than younger patients. These prescription patterns may be attributed to a lack of current evidence and concern about the adverse effects of statin use in dialysis patients, especially in elderly patients.

The present study also showed that the most widely used statin in dialysis patients with ASCVD in Korea was atorvastatin, accounting for approximately 60% of total statin use. The major issue of statin use in dialysis patients is the concern about the higher risk of side effects depending on decreased renal function. The renal side effects of statins can be associated with the extent of renal excretion. Atorvastatin has a limited issue because it has the least amount of renal excretion (< 2%) compared with other statins [17]. In addition, well-designed RCTs, such as 4D (the German Diabetes and Dialysis Study) [7] and TNT (Treating to New Targets) substudy [18], demonstrated the safety of atorvastatin in patients with CKD. The pharmacodynamics and previous RCTs for the safety of atorvastatin may have resulted in its major use in dialysis patients in Korea.

One noteworthy finding of this study was that the use of high-intensity statins and statin/ezetimibe combinations steadily increased in dialysis patients with ASCVD. Few studies on prescription patterns according to the statin intensity have shown that low- or moderate-intensity statins were mainly used in advanced CKD and ESRD patients after the 2000s [19]. The use of high-intensity statins in patients with CKD has been limited by concerns about the increased toxicity caused by reduced renal excretion, polypharmacy, and multiple comorbidities. Therefore, the 2013 KDIGO lipid guideline does not specify statin intensity recommendations [10]. However, the 2018 AHA/ACC and 2019 ESC/EAS cholesterol guidelines recommend that all patients with nondialysis-dependent CKD should be on moderate- to high-intensity statins in case of a high risk of ASCVD events [11, 12]. A recent meta-analysis showed that the adverse effects of high-intensity statins were not different from those of control groups in patients with CKD [20]. Additionally, the statin/ezetimibe combination was also found to be more effective than statin monotherapy in ASCVD patients with CKD [9, 21]. These factors have contributed to an increase in the use of high-intensity statins and statin/ezetimibe combinations in dialysis patients with ASCVD. Further studies may be needed to clarify the benefits and adverse effects of high-intensity statins in dialysis patients.

Previous landmark RCTs and meta-analyses have demonstrated that statins have little or no effect on the benefits for CV outcomes in dialysis patients [7–9, 22]. However, these RCTs included relatively low proportions of patients on dialysis who had a history of ASCVD, and up to one-third of primary composite outcomes included nonatherosclerotic CV events that may not be modified by statins. In contrast, *post hoc* analyses of RCTs have demonstrated that statins significantly reduced the risk of cardiac events in hemodialysis patients with a high risk of ASCVD [23, 24]. A recent observational study also demonstrated that statins are an independent predictor of a reduced risk of CV death in patients undergoing dialysis following percutaneous coronary intervention [25]. However, other observational studies did not demonstrate the benefits of statin therapy for CV outcomes in dialysis patients with ASCVD. A long-term observational study demonstrated a 30% reduction in all-cause mortality, but not the composite CV outcomes, in statin-treated patients with type 2 diabetes mellitus on dialysis after acute MI [26]. Chung et al. also reported that moderate- to high-intensity statins reduced the risk of all-cause mortality by 24% but did not influence CV outcomes in dialysis patients after acute MI during the 4-year follow-up [27].

Our study demonstrated that statin use was independently associated with reducing 1-year all-cause mortality in dialysis patients with ASCVD. In contrast, the use of statins was negatively associated with cardiac mortality, and this finding may reflect the result of the negative effect of statins in dialysis patients with PAD. In this study, statin use in patients with PAD was associated with a neutral effect on all-cause mortality but a deleterious effect on cardiac mortality. Although the mechanisms of the negative effects of statins in PAD cannot be elucidated, a recent study revealed that the use of statins was associated with PAD risk, which increased with cumulative statin dosage [28]. The plausible explanation for this effect was that the statins may accelerate vascular calcifications by inhibiting the synthesis of vitamin K2, which is a key regulator of vascular calcification [29]. Therefore, our results suggest that the appropriate dose and intensity of statins for the secondary prevention of ASCVD may differ, depending on ASCVD subtypes in dialysis patients.

The discrepancy between all-cause and cardiac mortality on statin use in dialysis patients could be explained by the following hypotheses. First, although CV disease is the major cause of death in dialysis patients, the pathogenesis of CV disease in ESRD is different from that of the general population. Several nontraditional risk factors, such as chronic volume overload, anemia, uremic toxins, and chronic kidney disease–mineral bone disorder (CKD–MBD), are implicated in the pathogenesis of CV disease in ESRD [30]. The influences of nontraditional factors may outweigh the importance of LDL-C levels and atherosclerosis in developing CV disease in dialysis patients. Therefore, the benefits of statin-based therapy may not be largely associated with decreased CV mortality in dialysis patients. Second, the pleiotropic effects of statins, such as the improvement of endothelial dysfunction, increased nitric oxide bioavailability, antioxidant properties, anti-inflammatory responses, and stabilization of atherosclerotic plaques [31], may reduce non-CV causes of death in dialysis patients. These findings suggest that the survival benefits of statins in ESRD may be associated with the pleiotropic effects of statins.

This study had several limitations. First, due to the nature of the observational, retrospective design, potential residual biases from measured or unmeasured confounders may have influenced the results. Second, we did not evaluate cause-specific deaths, except for cardiac mortality. Third, no information on smoking status, body mass index, socioeconomic status, or biochemical parameters, including lipid profile levels, hemoglobin, creatinine, phosphate, calcium, and albumin, was available. Therefore, the effects of survival on the achievement of the target goal of LDL-C or the parameters for CKD-MBD are difficult to know. Fourth, the enrolled patients comprised a homogeneous Asian population, and the generalizability to other ethnic groups may be limited. Last, this study could not validate the long-term mortality benefit of statin in dialysis patients after ASCVD events. A long-term follow-up study would be needed to address this issue. Despite these limitations, a strength of our study includes the large study population on a nationwide scale covering all dialysis patients in Korea. Our study is meaningful in realizing contemporary annual trends of statin prescription stratified by statin intensity and ASCVD subtypes in dialysis patients with new ASCVD events.

## Conclusion

We found that among dialysis patients with a first-time ASCVD event, statin use gradually increased from 2013 to 2018. Most patients received moderate-intensity statin; however, high-intensity statin and statin/ezetimibe combination therapy has remarkably increased. Statin therapy was associated with lowering 1-year all-cause mortality in dialysis patients after an ASCVD event. Additional studies further investigating the benefits of statins for the secondary prevention of ASCVD in dialysis patients may be needed.

## Supporting information

S1 Table Definitions of variables and outcomes. (DOCX)

S2 Table Baseline characteristics according to the ASCVD subtypes in dialysis patients. (DOCX)

S3 Table Trends of statin adherence in patients on dialysis with ASCVD from 2013 to 2018. (DOCX)

## Abbreviations

ASCVD: atherosclerotic cardiovascular disease
CCI: charlson comorbidity index
CHD: coronary heart disease
CKD: chronic kidney disease
CV: cardiovascular
CVA: cerebrovascular accident
ESRD: end-stage renal disease
MI: myocardial infarction
NHIS: national health insurance service
PAD: peripheral artery disease
PDC: proportion of days covered
RCT: randomized controlled trial
