# Supplementary material for: Trends and outcome of statin therapy in dialysis patients with atherosclerotic cardiovascular diseases: A population-based cohort study
Source: PLoS One. 2023 Jun 2;18(6):e0286670. doi: 10.1371/journal.pone.0286670 (PMC10237439; doi:10.1371/journal.pone.0286670)
Supplement: S1 Table — (DOCX) [file pone.0286670.s001.docx]

**Supporting Information File**

**Trends and outcome of statin therapy in dialysis patients with atherosclerotic cardiovascular diseases: A Population-Based Cohort Study**

Myunhee Lee ^1,2,*^, Yu Ah Hong ^3,*^, Jun-Pyo Myong ^4^, Kyusup Lee ^1,2^, Mahn-Won Park ^1,2^, and Dae-Won Kim ^1,2,†^

^1^ Division of Cardiology, Department of Internal Medicine, Daejeon St. Mary's Hospital, The Catholic University of Korea, Seoul, Korea; ^2^ Catholic Research Institute for Intractable Cardiovascular Disease CRID, College of Medicine, The Catholic University of Korea, Seoul, Korea; ^3^ Division of Nephrology, Department of Internal Medicine, Daejeon St. Mary's Hospital, The Catholic University of Korea, Seoul, Korea; ^4^ Department of Occupational and Environmental Medicine, Seoul St. Mary's Hospital, The Catholic University of Korea, Seoul, Korea

^*^ Myunhee Lee and Yu Ah Hong equally contributed to this work.

This appendix has been prepared by the authors to provide readers with additional information about their work.

**S1 Table. Definitions of variables and outcomes**

| **Variables** | **ICD-10 Code** | **Procedure/Diagnosis Code** | **Definition** |
| --- | --- | --- | --- |
| Chronic kidney disease / ESRD | N18, N181–N185, N189, N19, Z490, Z491, Z90, or Z905 |  |  |
| Hemodialysis |  | O7011–O7018, O7020, O7021 |  |
| Peritoneal dialysis |  | 321400BIS,321600BIS,321800BIS,324000BIS,324800BIS,325900BIS,326000BIS,349700BIS,349800BIS,349900BIS,350000BIS,350200BIS,350300BIS,352100BIS,352400BIS,352800BIS,365900BIS,366000BIS,366100BIS,366200BIS,366300BIS,423200BIS,423400BIS,423500BIS,431100BIS,431200BIS,431300BIS,449000BIS,449100BIS,449200BIS,449800BIS,449900BIS,450000BIS,463700BIS,463900BIS,464000BIS,464700BIS,464800BIS,494900BIS,495000BIS,495100BIS,509600BIS,509700BIS,509800BIS,510700BIS,510800BIS,511000BIS,513500BIS,644300BIJ,665700BIS,665800BIS,665900BIS |  |
| Angina | I20.x | HA524, HC292, HC297, HC298, HC301-5, EB434, EB435, E654, HC080-HC089, HA670 | (ICD-10 code) AND (Procedure or diagnosis code) AND (Admission or ER visit > 1 or outpatient clinic $\geq$ 2) |
| Myocardial infarction | I21.x – I22.x |  | (ICD-10 code) AND (Admission or ER visit > 1) |
| Coronary revascularization | I20-I25 | M6551, M6552, M6561-4, M6571, M6572, O1641, O1642, O1647, OA641, OA642, OA647 | (ICD-10 code) AND (Procedure code) AND (Admission or ER visit > 1) |
| Ischemic stroke | I63 | HA851, HA441, HA451, HA461, HE135, HE235, HE535, HA471, HE101, HE201, HE301, HE401, HE501 | (ICD-10 code) AND (Diagnosis code) AND (Admission or ER visit > 1) |
| Transient ischemic attack | G458, G459 | HA851, HA441, HA451, HA461, HE135, HE235, HE535, HA471, HE101, HE201, HE301, HE401, HE501 | (ICD-10 code) AND (Diagnosis code) AND (Admission or ER visit > 1), exclude if ICD-code for AF (I48.x) exist at index date |
| Peripheral artery disease | I70.2, I70.3, I70.9, I73.1, I73.8, I73.9 | M6597, M6605, M6613, M6632, M6620, O1643-4, O0161~O0171, O1645~6, N0572, N0573, N0574, N0575 | (ICD-10 code) AND (Diagnosis or Procedure code) AND (Admission or ER visit > 1 or outpatient clinic $\geq$ 2) |
| Cardiac mortality | I00 – I99 |  | Recorded as a major cause of death |

| Diagnosis for Charlson Comorbidity Index | Weight | ICD-10 Code |
| --- | --- | --- |
| Congestive Heart Failure | 1 | I43, I50, I099, I110, I130, I132, I255, I420, I425, I426, I427, I428, I429, P290 |
| Chronic Pulmonary Disease | 1 | J40, J41, J42, J43, J44, J45, J46, J47, J60, J61, J62, J63, J64, J65, J66, J67, I278, I279, J684, J701, J703 |
| Diabetes without complications | 1 | E100, E101, E106, E108, E109, E110, E111, E116, E118, E119, E120, E121, E126, E128, E129, E130, E131, E136, E138, E139, E140, E141, E146, E148, E149 |
| Diabetes with complications | 2 | E102, E103, E104, E105, E107, E112, E113, E114, E115, E117, E122, E123, E124, E125, E127, E132, E133, E134, E135, E137, E142, E143, E144, E145, E147 |
| Cancer | 2 | C00, C01, C02, C03, C04, C05, C06, C07, C08, C09, C10, C11, C12, C13, C14, C15, C16, C17, C18, C19, C20, C21, C22, C23, C24, C25, C26, C30, C31, C32, C33, C34, C37, C38, C39, C40, C41, C43, C45, C46, C47, C48, C49, C50, C51, C52, C53, C54, C55, C56, C57, C58, C60, C61, C62, C63, C64, C65, C66, C67, C68, C69, C70, C71, C72, C73, C74, C75, C76, C81, C82, C83, C84, C85, C88, C90, C91, C92, C93, C94, C95, C96, C97 |
| Moderate or Severe Liver Disease | 3 | K704, K711, K721, K729, K765, K766, K767, I850, I859, I864, I982 |
| Hypertension | 1 | I10 |
| Dyslipidemia | 1 | E78 |
| Atrial fibrillation | 1 | I48 |
