# Supplementary material for: Trends and outcome of statin therapy in dialysis patients with atherosclerotic cardiovascular diseases: A population-based cohort study
Source: PLoS One. 2023 Jun 2;18(6):e0286670. doi: 10.1371/journal.pone.0286670 (PMC10237439; doi:10.1371/journal.pone.0286670)
Supplement: S2 Table — (DOCX) [file pone.0286670.s002.docx]

**Supporting Information File**

**Trends and outcome of statin therapy in dialysis patients with atherosclerotic cardiovascular diseases: A Population-Based Cohort Study**

Myunhee Lee ^1,2,*^, Yu Ah Hong ^3,*^, Jun-Pyo Myong ^4^, Kyusup Lee ^1,2^, Mahn-Won Park ^1,2^, and Dae-Won Kim ^1,2,†^

^1^ Division of Cardiology, Department of Internal Medicine, Daejeon St. Mary's Hospital, The Catholic University of Korea, Seoul, Korea; ^2^ Catholic Research Institute for Intractable Cardiovascular Disease CRID, College of Medicine, The Catholic University of Korea, Seoul, Korea; ^3^ Division of Nephrology, Department of Internal Medicine, Daejeon St. Mary's Hospital, The Catholic University of Korea, Seoul, Korea; ^4^ Department of Occupational and Environmental Medicine, Seoul St. Mary's Hospital, The Catholic University of Korea, Seoul, Korea

^*^ Myunhee Lee and Yu Ah Hong equally contributed to this work.

This appendix has been prepared by the authors to provide readers with additional information about their work.

**S2 Table. Baseline characteristics according to the ASCVD subtypes in dialysis patients**

|  | **Overall (n=17242)** | **CHD**  **(n=11020)** | **CVA**  **(n=2867)** | **PAD**  **(n=3355)** | ***P* value** |
| --- | --- | --- | --- | --- | --- |
| **Index year** |  |  |  |  |  |
| 2013 | 2633 (15.3) | 1780 (67.6) | 507 (19.3) | 346 (13.1) | <.001 |
| 2014 | 2636 (15.3) | 1811 (68.7) | 441 (16.7) | 384 (14.6) | <.001 |
| 2015 | 2594 (15.0) | 1730 (66.7) | 445 (17.2) | 419 (16.2) | <.001 |
| 2016 | 3078 (17.9) | 1969 (64.0) | 497 (16.2) | 612 (19.9) | <.001 |
| 2017 | 3194 (18.5) | 1909 (59.7) | 537 (16.8) | 748 (23.4) | <.001 |
| 2018 | 3107 (18.0) | 1821 (58.6) | 440 (14.2) | 846 (27.2) | <.001 |
| **Age (year, %)** | 63.0 ± 11.3 | 62.7 ± 11.1 | 64.8 ± 11.1 | 62.5 ± 11.9 | <.001 |
| **Male sex (n, %)** | 10650 (61.8) | 6882 (62.5) | 1718 (59.9) | 2050 (61.1) | 0.02 |
| **Comorbidities (n, %)** |  |  |  |  |  |
| Diabetes | 11900 (69.0) | 7480 (67.9) | 2002 (69.8) | 2418 (72.1) | <.001 |
| Diabetes with chronic complications | 11561 (67.1) | 7439 (67.5) | 1946 (67.9) | 2176 (64.9) | 0.01 |
| Hyperlipidemia | 13245 (76.8) | 8969 (81.4) | 2024 (70.6) | 2252 (67.1) | <.001 |
| Hypertension | 16141 (93.6) | 10450 (94.8) | 2686 (93.7) | 3005 (89.6) | <.001 |
| Congestive heart failure | 7948 (46.1) | 5957 (54.1) | 920 (32.1) | 1071 (31.9) | <.001 |
| Atrial fibrillation | 1655 (9.6) | 1320 (12.0) | 0 | 335 (10.0) | <.001 |
| Chronic pulmonary disease | 6614 (38.4) | 4384 (39.8) | 1023 (35.7) | 1207 (36.0) | <.001 |
| Moderate to severe liver disease | 127 (0.7) | 79 (0.7) | 27 (1.0) | 21 (0.6) | 0.32 |
| Cancer | 1853 (10.8) | 1210 (11.0) | 284 (9.9) | 359 (10.7) | 0.25 |
| **CCI** |  |  |  |  |  |
| mean, SD | 4.93 ±1.74 | 5.09 ±1.73 | 4.62 ±1.67 | 4.67 ±1.78 | <.001 |
| CCI < 3, n (%) | 1630 (9.5) | 866 (7.9) | 350 (12.2) | 414 (12.3) | <.001 |
| CCI ≥ 3, n (%) | 15612 (90.5) | 10154 (92.1) | 2517 (87.8) | 2941 (87.7) | <.001 |

Data are mean ± SD or number (%).

ASCVD, atherosclerotic cardiovascular disease; CCI, charlson comorbidity index; CHD, coronary heart disease; CVA, cerebral vascular accident; PAD peripheral artery disease; SD, standard deviation.
