# Supplementary material for: Trends and outcome of statin therapy in dialysis patients with atherosclerotic cardiovascular diseases: A population-based cohort study
Source: PLoS One. 2023 Jun 2;18(6):e0286670. doi: 10.1371/journal.pone.0286670 (PMC10237439; doi:10.1371/journal.pone.0286670)
Supplement: S3 Table — (DOCX) [file pone.0286670.s003.docx]

**Supporting Information File**

**Trends and outcome of statin therapy in dialysis patients with atherosclerotic cardiovascular diseases: A Population-Based Cohort Study**

Myunhee Lee ^1,2,*^, Yu Ah Hong ^3,*^, Jun-Pyo Myong ^4^, Kyusup Lee ^1,2^, Mahn-Won Park ^1,2^, and Dae-Won Kim ^1,2,†^

^1^ Division of Cardiology, Department of Internal Medicine, Daejeon St. Mary's Hospital, The Catholic University of Korea, Seoul, Korea; ^2^ Catholic Research Institute for Intractable Cardiovascular Disease CRID, College of Medicine, The Catholic University of Korea, Seoul, Korea; ^3^ Division of Nephrology, Department of Internal Medicine, Daejeon St. Mary's Hospital, The Catholic University of Korea, Seoul, Korea; ^4^ Department of Occupational and Environmental Medicine, Seoul St. Mary's Hospital, The Catholic University of Korea, Seoul, Korea

^*^ Myunhee Lee and Yu Ah Hong equally contributed to this work.

This appendix has been prepared by the authors to provide readers with additional information about their work.

**S3 Table. Trends of statin adherence in patients on dialysis with ASCVD from 2013 to 2018**

|  | **2013**  **(n=1393)** | **2014**  **(n=1474)** | **2015**  **(n= 1455)** | **2016**  **(n= 1713)** | **2017 (n=1783)** | **2018 (n=1793)** | ***P* for trend** |
| --- | --- | --- | --- | --- | --- | --- | --- |
| **Overall (%)** | 73.1 | 75. 5 | 77.5 | 79.5 | 78.8 | 82.1 | 0.125 |
| **CHD (%)** | 76.9 | 78.9 | 80.1 | 82.8 | 81.6 | 86.0 | 0.004 |
| **CVA (%)** | 63.8 | 62.5 | 70.8 | 73.4 | 69.7 | 73.4 | 0.398 |
| **PAD (%)** | 62.0 | 67.8 | 69.0 | 70.1 | 75.4 | 75.0 | 0.647 |

Statin adherence was measured by the proportion of days covered during the 12 months after the index date.

CHD, coronary heart disease; CVA, cerebral vascular accident; PAD peripheral artery disease.
